# Chronic pain in the Chilean population: risk factors prevalence and cognitive associations

**DOI:** 10.3389/fragi.2025.1548667

**Published:** 2025-07-22

**Authors:** Hernán Hernández, Carolina Ochoa-Rosales, Agustín Ibáñez, Lorena Oyanadel, Loreto Olavarria, Nickole Marín-Díaz, Ariel Caviedes, Jessica L. Hazelton, Teresita Ramos, Hernando Santamaria-García, Nilton Custodio, Rosa Montesinos, Martin A. Bruno, Jose A. Avila-Funes, Diana Matallana, Rolando de la Cruz, Fanny Petermann-Rocha, Andrea Slachevsky, Claudia Duran-Aniotz, Carolina Gonzalez-Silva

**Affiliations:** ^1^ Latin American Institute for Brain Health (BrainLat), Universidad Adolfo Ibáñez, Santiago, Chile; ^2^ Department of Human Genetics, Radboud University Medical Center, Nijmegen, Netherlands; ^3^ Global Brain Health Institute, Trinity College, Dublin, Ireland; ^4^ Global Brain Health Institute, University of California San Francisco (UCSF), San Francisco, CA, United States; ^5^ Cognitive Neuroscience Center, Universidad de San Andrés and Consejo Nacional de Investigaciones Científicas y Técnicas, Buenos Aires, Argentina; ^6^ Specialist in Physical Medicine and Rehabilitation, Physical Medicine and Rehabilitation Service San Borja Arriaran Clinical Hospital, Pontificia Universidad Católica de Chile Health Network, Santiago, Chile; ^7^ Department of Psychiatry, Neuropsychology and Clinical Neuroscience Laboratory (LANNEC), Faculty of Medicine, Universidad de Chile, Santiago, Chile; ^8^ Escuela de Psicología, Facultad de Ciencias, Universidad Mayor, Las Condes, Chile; ^9^ Brain and Mind Centre, School of Psychology, The University of Sydney, Sydney, Australia; ^10^ Department of Neurology, Faculty of Medicine, Universidad Finisterrae, Santiago, Chile; ^11^ Memory Unit Neurology Service, Hospital del Salvador, Santiago, Chile; ^12^ Pontificia Universidad Javeriana (PhD Program in Neuroscience) Bogotá, San Ignacio, Colombia; ^13^ Center of Memory and Cognition Intellectus, Hospital Universitario San Ignacio Bogotá, San Ignacio, Colombia; ^14^ Instituto Peruano de Neurociencias, Unidad de diagnóstico de deterioro cognitivo y prevención de demencia, Lima, Peru; ^15^ Biomedical Science Institute, Faculty of Medicine, Universidad Católica de Cuyo, San Juan, Argentina; ^16^ Consejo Nacional de Investigaciones Científicas y Técnicas, Buenos Aires, Argentina; ^17^ L’Institut National de la Santé et de la Recherche Médicale (INSERM), Bordeaux Population Health Research Center, UMR 1219, University Bordeaux, Bordeaux, France; ^18^ Instituto Nacional de Ciencias Médicas y Nutrición Salvador Zubirán, Mexico City, Mexico; ^19^ Instituto de Envejecimiento, Facultad de Medicina, Pontificia Universidad Javeriana, Bogotá, Colombia; ^20^ Mental Health Department, Hospital Universitario Santa Fe de Bogotá, Bogotá, Colombia; ^21^ Faculty of Engineering and Sciences, Universidad Adolfo Ibáñez, Santiago, Chile; ^22^ Data Observatory Foundation, Santiago, Chile; ^23^ Centro de Investigación Biomédica, Facultad de Medicina, Universidad Diego Portales, Santiago, Chile

**Keywords:** Chile, chronic pain associated factors, cognitive impairment, elderly population, prevalence

## Abstract

Chronic pain (CP) is a global public health issue and a critical factor in the aging process. Chile, as one of the most aged countries in Latin America, presents a unique context for exploring CP and its associated factors. Despite its significance in aging, previous studies in the region often fail to comprehensively address key variables such as age, income, mood, mobility, diet, and cognitive skills, nor do they systematically investigate the relationship between CP and cognitive impairment. This study presents a comprehensive analysis of CP prevalence, related sociodemographic and health variables, and its link to cognitive impairment, using representative data of the Chilean population 15 years and older from the 2009–2010 and 2016–2017 Chilean National Health Surveys (CNHS). In the expanded sample of 12,791,542 and 13,399,937 individuals respectively, the overall prevalence of CP was 46.0% in the 2009–2010 CNHS and 28.9% in the 2016–2017 CNHS, with prevalence increasing with age. CP ranged from 26.6% among individuals aged 15–24 years to 59.9% among those aged 65–80 years in the 2009–2010 CNHS, and from 16.6% to 40.2% in the 2016–2017 CNHS. Female participants consistently reported higher CP rates, with significantly higher prevalence than males across age groups 15–64 years. Using complex survey logistic regression analyses, we identified several factors that were significantly associated with CP, including reduced mobility, depression, anxiety, socioeconomic disadvantage, and lower educational attainment. Machine learning techniques were employed to classify CP and non-CP cases, providing a nuanced understanding of the complex interplay between factors that influence CP. In a secondary analysis among those 60 years and older, no significant difference in CP prevalence was observed between individuals with and without cognitive impairment measured with an abbreviated MiniMental State Examination test. However, those with cognitive impairment tended to report pain in a greater number of anatomical sites. This study provides the first nationally representative evidence of CP in Chile in relation to age, income, mood, mobility, diet, and cognitive performance. These findings contribute to the understanding of CP as a public health issue in Latin America. The study underscores the need for targeted interventions to promote healthy longevity and reduce the burden of chronic diseases in aging populations.

## 1 Introduction

Chronic pain (CP) is characterized by pain persisting beyond the expected healing period or lasting for more than 3 months (Raja et al., 2020). Unlike acute pain, which typically arises in response to a discernible noxious stimulus, CP can manifest and persist in the absence of apparent harm (Costigan et al., 2009). The global burden of CP is substantial, affecting approximately 28% of the worldwide population, with prevalence increasing notably with age (Zimmer et al., 2022). From an economic perspective, excluding institutionalized individuals, the cost of CP is estimated to range between 560 and 635 billion dollars annually due to medical expenses and productivity losses in America (Cohen et al., 2021).

Throughout Latin America (LA), CP prevalence varies significantly across regions (Castañeda Morales et al., 2015; Lasalvia et al., 2022; Leyva et al., 2023; Martineau et al., 2023; Oliveira et al., 2023; Serra et al., 2021; Souza et al., 2017). Although studies in Chile report CP prevalence between 32.1% and 34.7% (Bilbeny et al., 2018; Durán et al., 2021), these studies often have limitations and lack representativeness. Specifically, previous studies have frequently overlooked key demographic and socioeconomic factors unique to the population and fail to fully capture CP prevalence within the aging demographic.

The high prevalence reported in LA and Chile could be attributed to identified risk factors, including age, female sex, low education level, and depressive symptoms (Oliveira et al., 2023; Durán et al., 2021; De Moraes Vieira et al., 2012; Durán et al., 2023; Leão et al., 2016; Maia Costa Cabral et al., 2014). Additionally, CP is associated with impairments in daily activities, malnutrition, sleep disorders, anxiety, depression, suicidal ideation, and suicidal behavior (Durán et al., 2023; Maia Costa Cabral et al., 2014; Dellaroza et al., 2013; Martínez et al., 2016; Morete et al., 2018; Paz et al., 2021; Pereira et al., 2017; Schmiesing et al., 2022; Silvestre et al., 2023; Vélez et al., 2022). Pronounced social inequality also affects the perception and occurrence of CP, highlighting the significant influence of environmental factors in CP (Ibanez et al., 2024; Adkins‐Jackson et al., 2023; Bartoskova Polcrova et al., 2023; Deguen et al., 2022). CP has also been associated with pathological aging (Pereira et al., 2017; Cao et al., 2019; Chen et al., 2023; Innes and Sambamoorthi, 2020; Moriarty et al., 2011; Phelps et al., 2021; Takahashi et al., 2018; Whitlock et al., 2017). Emerging evidence, particularly from high-income countries and based on extensive cohort studies, suggest that greater CP is associated with cognitive decline (Whitlock et al., 2017; Milani et al., 2023; Milani et al., 2024; Nahin and DeKosky, 2020). This relationship, however, has not been systematically explored in LA (Morete et al., 2018; Milani et al., 2023; Milani et al., 2024; Migeot et al., 2024; Prado et al., 2023; Santamaria-Garcia et al., 2023). Furthermore, despite the biological and social diversity in Chile and the high prevalence of CP in the country (Bilbeny et al., 2018; Durán et al., 2023), the factors associated with CP that are specific to this context have not been thoroughly explored. In addition, the relationship between CP and cognitive impairment among older Chilean adults remains unexamined.

Firstly, this study aimed to assess the prevalence of CP and its association with key demographic, socioeconomic, and health-related variables; secondly, it sought to explore the potential relationship between CP and suspected cognitive impairment (hereinafter referred to as cognitive impairment) among older adults. We hypothesized that CP in Chile is associated with specific regional adverse socioeconomic (Oliveira et al., 2023; Fittipaldi et al., 2024; Bonathan et al., 2013; Deckers et al., 2019) and health conditions (Ibanez et al., 2024; Nahin and DeKosky, 2020; Migeot et al., 2024; Santamaria-Garcia et al., 2023). Further, we anticipated that these factors would vary based on sex and age (Cohen et al., 2021; Oliveira et al., 2023; Serra et al., 2021).

## 2 Materials and methods

### 2.1 Study design

This cross-sectional study was based on two nationally representative cycles of the Chilean National Health Survey (CNHS): 2009–2010 (Min istry of Health MINSAL, 2010) and 2016–2017 (Ministry of Health MINSAL, 2017). These prevalence studies are surveys conducted by the Ministry of Health to assess the population’s health status and associated sociodemographic and clinical factors. Both surveys employed a stratified, multistage probability sampling design to ensure representativeness of the Chilean population 15 years and older. We selected these two specific survey waves because they are the most recent to include harmonized data on CP, cognitive function, mood disorders, mobility, nutritional status, and socioeconomic conditions. The protocol was approved by the Research Ethics Committee of the Pontificia Universidad Católica de Chile (No. 16–019) and authorized by the Public Health Subsecretariat. Written informed consent was obtained from all participants, in accordance with the Declaration of Helsinki. Supplementary Figure 1 displays the data processing pipeline, including dataset selection, feature extraction, outcome definitions, statistical approaches, classifier details, and performance results.

### 2.2 Study population

The CNHS cross-sectional studies conducted in 2009–2010 and 2016–2017 surveyed a total of n = 5,290 and n = 6,051 individuals aged 15 years and older, respectively. In the current study, we included participants with available data on self-reported CP, comprising n = 4,683 for the CNHS 2009–2010 and n = 4,887 for the CNHS 2016–2017, corresponding to an expanded population of 12,791,542 and 13,399,937 respectively (Supplementary Figure 1A). Exclusion criteria considered individuals older than 80 years (n = 190 in 2009–2010 and n = 274 in 2026–2017) to prevent outliers, those who reported cancer diagnosis (n = 30 in 2009–2010 and n = 243 in 2026–2017) to focus on non-cancer CP, and those with missing data on the analytical variables (n = 387 in 2009–2010 and n = 647 in 2026–2017). Additional analysis on the relationship between CP and cognitive impairment was conducted among a sample of n = 1,104 (2009–2010, expanded population of 2,049,500), and the n = 1,401 (2016–2017, expanded population of 2,214,309) adults 60–80 years. Overall, both surveys were selected due to their population representativeness, the inclusion of a large number of individuals living with CP and the comprehensive clinical and sociodemographic data available.

### 2.3 Variables

Data in the 2009–2010 and 2016–2017 CNHS were collected during home interviews by trained interviewers. The same methods were used across both surveys.

#### 2.3.1 Outcome variables

##### 2.3.1.1 Chronic pain

In this study the presence of CP was classified dichotomously identifying whether participants had self-reported none-cancer CP. Non-cancer CP was defined as an unceasing pain lasting more than 3 months or a pain that persists beyond the normal healing time, which usually has no protective function, impairs health, and causes substantial disability (Treede et al., 2019). In addition, participants were interviewed regarding the joints where they experienced pain in the past weeks and the duration of this pain. Further, data were collected on the number of anatomical sites in which participants reported feeling pain, including the neck, shoulder, upper back, lower back, elbow, wrist, fingers, hip, knee, ankle, and toes. Single-site pain referred to pain localized to a specific area of the body, while multiple-site pain encompassed pain experienced across multiple body regions.

##### 2.3.1.2 Cognitive function

Among those 60 years and older, an abbreviated validated Chilean version of the Mini-Mental State Examination (MMSE) instrument was used to assess suspected cognitive function. The MMSE measures cognitive domains including orientation, attention, recent memory, and language (Folstein et al., 1975). This version comprises six questions with a possible score ranging from 0 to 19. A score 13 or lower was considered as evidence of cognitive impairment (Icaza and Albala, 1999). For this analysis, the MMSE score and the number of painful sites were treated as binary outcomes based on established cutoffs (≤13).

#### 2.3.2 Predictors

##### 2.3.2.1 Demographics

Demographic features included sex (female or male, no questions regarding gender identity were made), educational attainment (years of education completed) and age.

##### 2.3.2.2 Socioeconomic status (SES)

Data on access to electricity, potable water, sanitation facilities, cooking fuel, and household equipment was collected. Further, a SES score ranging from 0 to 10 was created, assigning one point for each positive condition and calculated a percentage based on 10, where 10 signifies the maximum score assigned to homes with optimal conditions (Santamaria-Garcia et al., 2023).

##### 2.3.2.3 Mood disorders

Mood disorders covered in the study consisted of self-reported cases of anxiety and depression among the participants, based on data from the psychosocial module. Participants were asked about the frequency of their feelings of anxiety or stress, rated on a four point Likert scale raining from. i) never, ii) sometimes at home or work, iii) several times at home or work, or iv) permanently at home or work. Individuals who reported feeling stress on a permanent basis or several times throughout the week were classified as suffering from anxiety. Participants were also asked to rate their emotional state on a three point scale: i) I am not distressed or depressed, ii) I am moderately distressed or depressed, or iii) I am very distressed or depressed. Individuals who reported being moderately or very distressed or depressed were classified as suffering from depression.

##### 2.3.2.4 Nutritional status

The Body Mass Index (BMI) in the survey was derived as body weight divided by height squared, measured by a trained nurse/interviewer. Subjects having a BMI lower than 18.5 classified as underweight (malnutrition), those with BMI 18.6 to 24.9 as normal weight, those with BMI 25 to 29.9 are overweight, while a BMI greater than 30 classified as obese (Nuttall, 2015).

##### 2.3.2.5 Mobility reduction

The assessment of mobility was based on participants’ reports of their limitations in proper movement. Subjects are asked if they have difficulty walking, required assistance to move around or if they have to stay at home due to their walking problems.

### 2.4 Statistical analyses

The characteristics of the study population were described using means and standard deviations (SD) for numerical variables with a normal distribution, and medians with interquartile ranges (IQR) for those not normally distributed. For categorical variables, absolute and relative frequencies were used. Furthermore, the prevalence of CP was calculated using an expansion factor, as indicated in the survey’s methodological analysis manual, and stratified by sex and age groups: a) 15–24 years, b) 25–44 years, c) 45–64 years, and d) 65–80 years.

To assess the association between the presence of CP (dependent variable) and lower mobility, nutritional status, and self-reported mood disorders (independent variables), we used complex survey logistic regression models, adjusting for SES, education, sex, age, and geographical region. Next, we examined the association between CP and both education and SES, using sex, age, and geographical region as covariates. Results were expressed as odds ratios (ORs) and 95% confidence intervals (95% CI) (Andrade, 2015; Szumilas, 2010), and analyses were conducted separately for each survey. An OR greater than one indicates higher odds of the event (presence of CP) occurring in the reference group; an OR less than one indicates lower odds; and an OR equal to one suggests no association.

In the secondary analysis among those 60–80 years, we used the presence of cognitive impairment (yes/no) as the dependent variable to study the association with the presence of CP and with the number of pain sites. For this, we conducted complex survey logistic regression models, adjusting for SES, education, sex, age, and geographical region within the country.

To evaluate the capacity for distinguishing between CP and non-CP cases across the whole sample, we employed the XGBoost classification algorithm (Chen and Guestrin, 2016). The analyses were conducted separately for each survey and were further stratified by cognitive impairment status. XGBoost is a machine learning algorithm that has emerged as a high-performance alternative (Chen and Guestrin, 2016; Sagi and Rokach, 2018). Based on extreme gradient boosting principles, it has consistently outperformed in various applications (Sagi and Rokach, 2018; Gündoğdu, 2023). Models were trained on an 80% training sample and subsequently tested on a 20% testing set, with k = 10 repetitions (Müller and Guido, 2016). For each iteration, the f-score for the features, Area Under the Curve (AUC), accuracy, precision, f1, and recall were computed. Mean f-scores for each feature, along with their standard deviations, were reported. Bayesian optimization was employed to identify the optimal hyperparameters for XGBoost. By delineating a search space for hyperparameters, employing a surrogate probabilistic model, and iteratively proposing new configurations, Bayesian Optimization efficiently explores the parameter space (Shahriari et al., 2016). By introducing XGBoost models, we leveraged a powerful machine learning algorithm capable of capturing complex patterns and interactions within the data, which traditional logistic regressions might overlook. XGBoost also allows for better handling of large and unbalanced datasets, improving the overall model performance. The ability to optimize hyperparameters using Bayesian optimization further enhances the precision and accuracy of the predictions, providing a more comprehensive analysis compared to logistic regressions alone. All analyses were conducted in Python (v3.12.4) using the statsmodels, scikit-learn, and XGboost packages, and Stata (SB v18.0) (Müller and Guido, 2016; Python Software Foundation, 2024).

## 3 Results

A baseline description of the study population’s characteristics is presented in Table 1. The mean [SD] age of respondents in the 2009–2010 survey was 44.87 [17.4] years (female: 45.06 [17.2], male: 44.85 [17.6]), while in the 2016–2017 survey it was 46.88 [18.0] years (female: 47.27 [17.8], male: 46.23 [18.4]). The mean number of years of education in 2009–2010 was 9.88 [4.1] years (female: 9.79 [4.2], male: 10.03 [4.1]), and in 2016–2017 it was 10.35 [4.1] years (female: 10.17 [4.1], male: 10.64 [4.1]).

**TABLE 1:** Demographic, education, and chronic pain data from the CNHS 2010 and CNHS 2016 Surveys.

CNHS survey	Number of analyzed subjects	Representative sample	Average of age (SD)	Average of education years (SD)	Subjects suffering CP (%)	Subjects older than 60 years old	Subjects older than 60 years old with cognitive impairment
2009–2010	4,683	12,791,542	44.87 (17.4)	9.88 (4.1)	2,179	1,104	90
	F: 59.3%		F: 45.06 (17.2)	F: 9.79 (4.2)	F: 64.9%	F: 56.5%	F: 44.4%
	M: 40.7%		M: 44.85 (17.6)	M: 10.03 (4.1)	M: 35.1%	M: 41.5%	M: 55.6%
2016–2017	4,887	13,399,937	46.88 (18.0)	10.35 (4.1)	1,447	1,401	133
	F: 62.2%		F: 47.27 (17.8)	F: 10.17 (4.1)	F: 69.6%	F: 62.1%	F: 57.1%
	M: 37.8%		M: 46.23 (18.4)	M: 10.64 (4.1)	M: 30.4%	M: 37.9%	M: 42.9%

In regard to cognitive status among those 60–80 years old, 90 out of/1,104 (2009–2010 survey) and 133 out of/1,401 (2016–2017) participants presented with cognitive impairment. in those aged 60 and over.

### 3.1 Chronic pain prevalence in Chile

In the 2009–2010 survey, 2,179 subjects reported experiencing CP (female: 64.9%), while in the 2016–2017 CNHS, 1,447 subjects reported suffering from CP (female: 69.6%).

**FIGURE 1:**
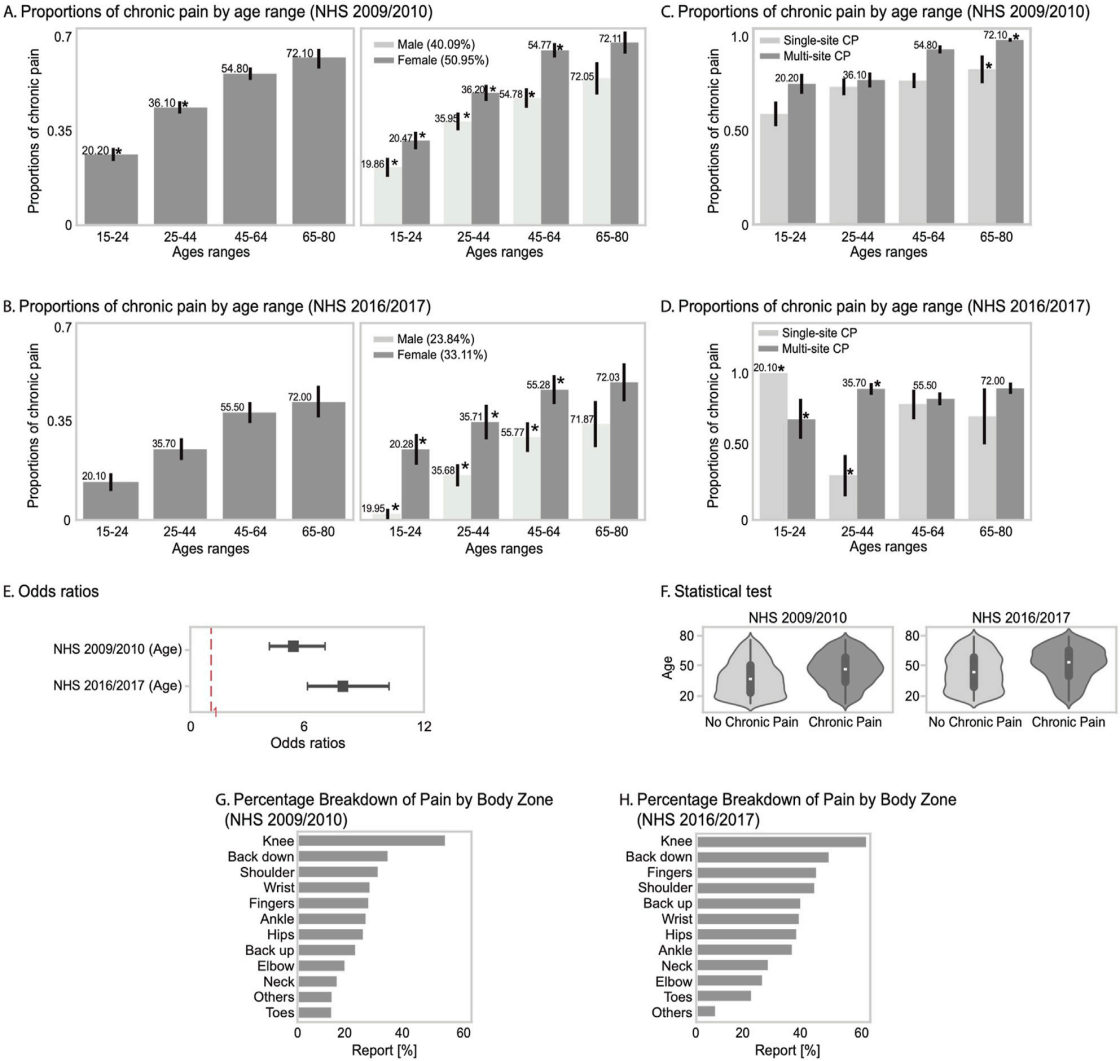
Panels **(A)** and **(B)** show the proportion of individuals reporting chronic pain by age group in the NHS 2009/2010 and 2016/2017 surveys, disaggregated by sex with paired bars and significant differences indicated. Panels **(C)** and **(D)** display proportions of individuals with single- vs. multi-site chronic pain across age groups. Panel **(E)** presents odds ratios of chronic pain by age for both survey waves with 95% confidence intervals. Panel **(F)** includes violin plots of age distributions by chronic pain status. Panels **(G)** and **(H)** show the most frequently reported painful body zones for each survey year.

Furthermore, CP prevalence tended to be higher in older age groups, with the highest age-specific prevalence observed among individuals aged 65–80 years. Specifically, this group showed a prevalence of 59% (95% CI: 52%–66%) in the 2009–2010 survey and 42% (95% CI: 31%–53%) in the 2016–2017 survey (Figures 1A, B, left panels; Supplementary Table 1). Notably, women reported higher CP rates than men across all age ranges in both surveys. These sex differences were statistically significant in the 15–64 age range (p < 0.05) but not in the 65–80 age group, where the differences were no longer significant.

Women reported a higher proportion of CP than men across all age groups in both the 2009–2010 and 2016–2017 surveys (Figures 1A, B, right panels; Table 2).

**TABLE 2:** Proportions of CP by age range and split by sex, for the 2009–2010 and 2016–2017 CNHS surveys.

Age range (years)	Sex	CNHS 2009–2010		CNHS 2016–2017	
		Proportion	95% conf. interval	Proportion	95% conf. interval
15–24	Male	0.20	0.14–0.28	0.02	0.004–0.11
	Female	0.30	0.24–0.36	0.25	0.16–0.37
25–44	Male	0.37	0.31–0.43	0.16	0.10–0.25
	Female	0.47	0.41–0.53	0.35	0.24–0.48
45–64	Male	0.45	0.38–0.52	0.30	0.20–0.41
	Female	0.62	0.57–0.67	0.46	0.37–0.57
65–80	Male	0.52	0.41–0.63	0.34	0.20–0.52
	Female	0.65	0.57–0.72	0.49	0.36–0.62

Moreover, regarding the number of body pain sites, in the 2009–2010 survey, individuals aged 65–80 years were more likely to experience multi-site CP than single-site CP (Figure 1C; Supplementary Table 2). To explore whether the observed age-related patterns in CP extend beyond body site distribution, we performed a trend analysis examining the general association between age and CP. We found a significant positive relationship in both 2009–2010 (OR = 7.80 [5.98–10.18]) and 2016–2017 (OR = 5.24 [4.00–6.88]) surveys. This association remained statistically significant despite a slight decrease in magnitude over time. Additionally, age distributions differed significantly between individuals with and without CP (p < 0.0001), with moderate effect sizes in both periods (d = 0.49 and d = 0.41). These findings provide further evidence that age plays a stable and substantial role in CP prevalence across aging (Figures 1E, F).

A more detailed analysis of specific pain locations in the 2009–2010 survey reveals that knee and low back pain were the most commonly affected areas in the Chilean population (Figure 1G; Supplementary Table 3), and this pattern persisted in the 2016–2017 survey (Figure 1H; Supplementary Table 3).

### 3.2 Main factors associated with CP in Chile

**FIGURE 2:**
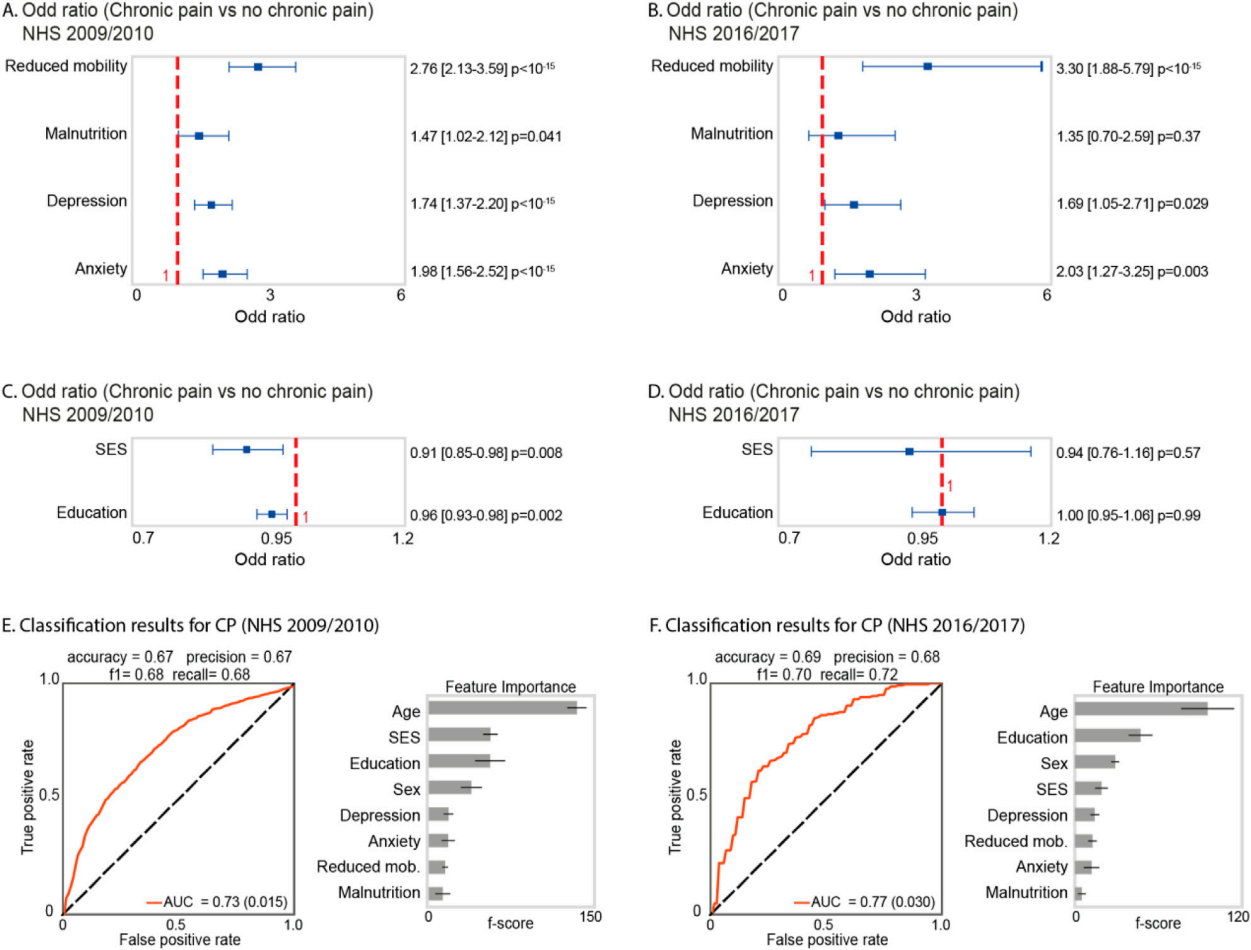
Statistical Analysis and Classification Results. **(A)** and **(B)** show the OR results and 95% confidence intervals for four variables: reduced mobility, malnutrition, depression, and anxiety in relation to the presence of CP for the CNHS 2009–2010 and 2016–2017 surveys, respectively. **(C)** and **(D)** display the OR results and 95% confidence intervals for SES and education in relation to CP for the same surveys. **(E)** and **(F)** show in the left panel the classification results using XGBoost for CP, and in the right panel the predictor importance in classification.

Our analysis demonstrated that reduced mobility emerges as the most pronounced factor linked with CP across both the 2009–2010 and 2016–2017 surveys, with an OR of 2.76 [2.13–3.59] and 3.30 [1.88–5.79], respectively (Figures 2A, B). Similarly, greater anxiety was consistently associated with presence of CP in both survey periods (Figures 2A, B). Additionally, the presence of depression was significantly associated with CP in the 2009–2010 survey only (Figure 2A). Furthermore, no association was observed with nutritional status in either survey (Figures 2A, B).

Moreover, we observed that individuals with higher SES (OR: 0.91 [0.85–0.98]) and higher educational attainment (OR: 0.96 [0.93–0.98]) were less likely to report presence of CP compared to those with lower SES and educational levels in the 2009–2010 survey (Figure 2C), respectively. Contrarily, no significant associations were observed for the 2016–2017 survey (p values ≥ 0.05) (Figure 2D).

The classification analysis aimed at identifying primary factors associated with CP in the 2009–2010 and the 2016–2017 CNHS surveys emphasized age as the predominant risk factor in both survey periods (Figures 2E, F). Additionally, sex, education, and SES exhibited significant association with CP occurrence among the Chilean population (Figures 2E, F). Notably, self-reported mental mood disorders such as anxiety and depression also emerged as factors associated with CP. Malnutrition (unhealthy weight) demonstrated the lowest frequency in CP presence (Figures 2E, F). Additionally, in the CNHS 2009–2010 survey, we achieved the following classification performance for CP vs. non-CP cases AUC = 0.73 (0.015), accuracy = 0.67, precision = 0.67, f1-score = 0.68, and recall = 0.68. Moreover, for the 2016–2017 survey, the results were as follows: AUC = 0.77 (0.030), accuracy = 0.69, precision = 0.68, f1-score = 0.70, and recall = 0.72.

### 3.3 Chronic pain and its association with cognitive impairment

**FIGURE 3:**
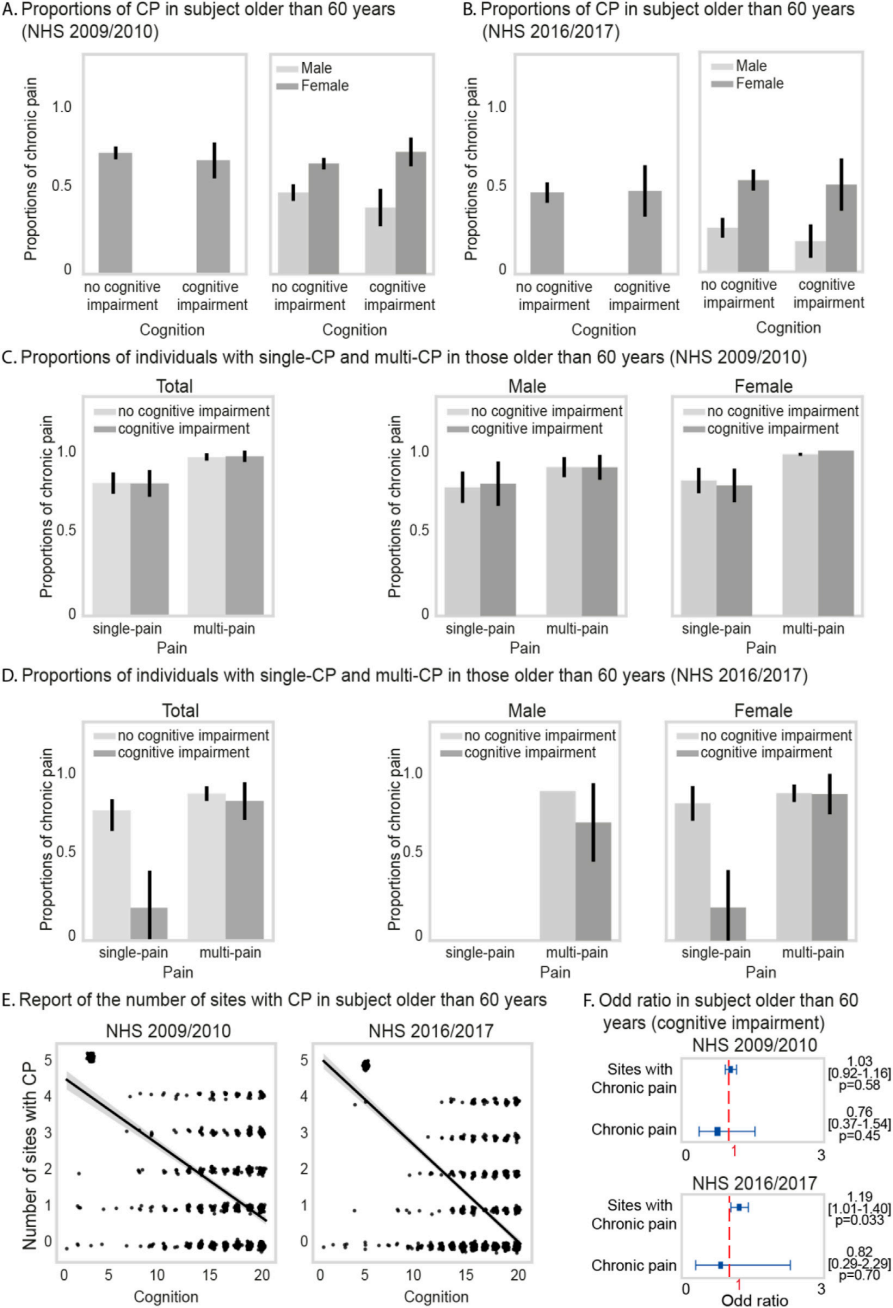
Panels **(A)** and **(B)** display the proportion of chronic pain (CP) among individuals with and without cognitive impairment using CNHS 2009–2010 and 2016–2017 data. Panels **(C)** and **(D)** show the proportion of single-site and multi-site CP in both groups across surveys. Panel **(E)** illustrates a linear relationship between the number of pain sites and cognitive performance. Panel **(F)** presents odds ratios and 95% confidence intervals for the association between cognitive impairment and both the number of pain sites and presence of CP, based on data from both CNHS survey periods.

In the 2009–2010 survey, the prevalence of CP in individuals aged 60–80 years was comparable between those with normal cognitive function (58% [95% CI: 52%–64%]) and those experiencing cognitive impairment (54% [95% CI: 38%–0.70%]) (Figure 3A, left panel. Table 3). Importantly, in sex-specific analysis among individuals with cognitive impairment, female participants showed a slightly higher prevalence of CP (73% [95% CI: 53%–87%]), as compared to males (40% [95% CI: 21%–62%]) (Figure 3A, right panel; Table 3).

**TABLE 3:** Proportions of CP among individuals aged 60 and older, segmented by sex, for the CNHS surveys conducted in 2009–2010 and 2016–2017.

Cognition	Sex	CNHS 2009–2010		CNHS 2016–2017	
		Proportion	95% conf. interval	Proportion	95% conf. interval
No-cognitive impairment	Total	0.58	0.52–0.64	0.39	0.30–0.49
	Male	0.49	0.39–0.58	0.26	0.16–0.39
	Female	0.66	0.59–0.72	0.55	0.42–0.67
Cognitive impairment	Total	0.54	0.38–0.70	0.40	0.19–0.65
	Male	0.40	0.21–0.62	0.18	0.06–0.47
	Female	0.73	0.53–0.87	0.52	0.23–0.79

Subsequently, in the analysis of the CNHS spanning 2016–2017, the prevalence of CP among the same age cohort was 40% [95% CI: 19%–65%] for participants with cognitive impairment and 39% [95% CI: 30%–49%] for those with normal cognition (Figure 3B, left panel; Table 3). Consistent with the 2009–2010 survey, a slightly higher prevalence of CP was found in female participants with cognitive impairment (52% [95% CI: 23%–79%]), as compared to males with the same cognitive state 18% [95% CI: 6%–47%]) (Figure 3B, right panel; Table 3).

Furthermore, the 2009–2010 survey showed comparable prevalence of single-site and multi-site CP among individuals with normal cognition and those with cognitive impairment (Figure 3C; Supplementary Table 4). Similarly, the 2016–2017 survey indicates consistent prevalence for both cognitive states (Figure 3D; Supplementary Table 4). No significant differences were observed between female and male and subjects reporting single-site or multi-site CP across cognitive states.

Finally, analysis between the MMSE score and the number of painful sites, revealed that individuals with cognitive impairment tended to experience pain in a greater number of anatomical sites (Figure 3E, left and right panels). Specifically, in the 2016–2017 CNHS data, individuals with cognitive impairment were 1.19 [1.01–1.40] times more likely to report pain in various anatomical sites than those with normal cognition (Figure 3F, lower panel). No significant results were found in the 2009–2010 survey (Figure 3F, upper panel).

## 4 Discussion

In a representative sample, we investigated the prevalence of CP in the Chilean population using data from the 2009–2010 and 2016–2017 CNHS. Our analyses revealed a CP prevalence of 46.5% (female: 64.9%) in the 2009–2010 survey and 29.6% (female: 69.6%) in the 2016–2017 survey, in participants 60–80 years.

Age-specific analysis revealed a higher prevalence of CP with increasing age, peaking at 59% and 42% in the 65–80-year age group for the 2009–2010 and 2016–2017 survey periods, respectively.

Additionally, our findings confirmed a consistently higher prevalence of CP among females across all age groups in both survey periods. These sex differences were statistically significant among individuals aged 15–64 years (p < 0.05) but not in those aged 65–80 years, where prevalence levels between sexes converged. This pattern suggests that gender disparities in CP are more pronounced during early and mid-adulthood and tend to diminish in older age. This attenuation may be explained by the general increase in CP prevalence with aging across both sexes, which has been widely reported in the literature and may reduce the relative difference between males and females in later life (Castañeda Morales et al., 2015; Serra et al., 2021; Morete et al., 2018). Structural barriers—such as limited access to specialized pain management services, unequal healthcare utilization, and entrenched gender roles—may influence the experience and reporting of pain among women across the lifespan (Oliveira et al., 2023; Silvestre et al., 2023; Bonathan et al., 2013). However, cultural norms and gendered expectations regarding health-seeking behaviors likely have a stronger impact in younger and working-age populations, contributing to the observed sex differences in the 15–64 age range (Befus et al., 2018; Meghani et al., 2012). Across both surveys, the most commonly affected anatomical sites remained the lower back and knees, consistent across age groups. These findings mirror global patterns and reflect the dual influence of occupational exposures (more prevalent in younger adults) and age-related musculoskeletal degeneration, such as osteoarthritis, in older individuals (Takahashi et al., 2018; Leme et al., 2019).

The logistic regression analysis provided critical insights into the determinants of CP in the Chilean population. Our findings indicated that reduced mobility was the most significant factor associated with CP in both the 2009–2010 and 2016–2017 surveys, underscoring the critical importance of functional status in the experience of pain. This relation aligns with recent research that reported that the decreased range of motion and mobility limitations were strongly associated with increased pain and disability in older adults and those with conditions like knee osteoarthritis (Edwards et al., 2022).

Anxiety also emerged as a significant factor, emphasizing the interplay between mental health and CP in Chile. This association is well-documented in other regions, with numerous studies indicating that anxiety and other psychological factors are crucial determinants of CP experiences (Arango-Dávila and Rincón-Hoyos, 2018; Asmundson and Katz, 2009; Jennifer et al., 2024; Kawai et al., 2017; Lerman et al., 2015). The consistent identification of these factors across different survey periods accentuate the necessity for integrated management strategies that address both physical and mental health dimensions. Depression was significantly associated with CP in the 2009–2010 survey but not in the 2016–2017 survey. Previous research has shown that CP and depression often co-occur, with each condition potentially exacerbating the other (Arango-Dávila and Rincón-Hoyos, 2018; Jennifer et al., 2024; Kawai et al., 2017; Lerman et al., 2015; Aguilar-Latorre et al., 2023). The discrepancy between the results that we observed in depression and CP in the 2009–2010 and 2016–2017 survey may suggest potential changes in the mental health landscape or reporting practices over time. Taken together, our results highlight the multifaceted nature of CP in the relation to both physical and mental health in Chile.

Notably, our findings from the 2009–2010 survey indicate that individuals with higher SES and education levels had a lower likelihood of reporting CP. However, this finding was not observed in the 2016–2017 survey. This might reflect evolving socioeconomic conditions in Chile or differences in the population samples. Notably, while aging, lower educational attainment, and female sex have previously been recognized as risk factors associated with CP in LA (Oliveira et al., 2023; Durán et al., 2021; De Moraes Vieira et al., 2012; Durán et al., 2023; Leão et al., 2016; Maia Costa Cabral et al., 2014; Pereira et al., 2014), the identification of a relationship between SES and CP in Chile is a novel contribution of this study. Our findings highlight the necessity for addressing, in addition to physiological aspects of pain, the broader socioeconomic determinants that contribute to CP, particularly in regions characterized by pronounced levels of inequality, such as LA (Migeot et al., 2024; Prado et al., 2023; Santamaria-Garcia et al., 2023; Fittipaldi et al., 2024; Baez et al., 2023; Kalaria et al., 2024). Indeed, previous studies have shown that socioeconomic disparities can significantly influence health outcomes, including the prevalence and management of CP (Williams and Mohammed, 2009; Yong et al., 2022). For instance, lower SES is often associated with increased stress, limited access to healthcare, and fewer resources for effective pain management, which can exacerbate the experience of CP (Wallace et al., 2021; Zitko et al., 2021).

In line with the aforementioned, differences in CP prevalence in the overall sample from 2009 to 2010 survey (45.5%) and 2016–2017 survey (29.6%) may find a partial explanation in the substantial health and social policy reforms implemented during this period. These included the expansion of the Universal Access with Explicit Guarantees (AUGE) program and the strengthening of primary healthcare services, which improved access to pain and mental health management (Superintendencia de Salud, 2025). Supporting this interpretation, national statistics from the National Socioeconomic Characterization Survey (CASEN) document a reduction in poverty rates among older adults and an increase in average educational attainment—both of which are known to influence CP outcomes (Ministry of Social Development and Family, 2018). These structural improvements may have attenuated the association between CP and socioeconomic disadvantage observed in the earlier survey. Our findings also reaffirmed that CP in Chile is strongly associated with older age, female sex, lower educational attainment, and limited financial resources. To our knowledge, this is the first study to characterize this constellation of factors using robust, population-level data in the Chilean context. While overall CP prevalence was not significantly different between individuals with and without cognitive impairment, we found that those with cognitive impairment were more likely to report multi-site pain in the 2016–2017 cohort. This finding may be due to a smaller number of participants who had both cognitive testing and CP measures available and may also reflect the cross-sectional nature of our study. Indeed, previous longitudinal studies have revealed a bidirectional relationship between CP and cognitive impairment, where initial CP predicts subsequent cognitive impairment which then predicts further CP, adding complexity to understanding the CP-dementia relationship (Cao et al., 2019; Chen et al., 2023; Innes and Sambamoorthi, 2020). When examining cognition in more depth, we found that worse cognitive performance was associated with greater multi-site pain, however, this was restricted to the 2016–2017 CNHS where data was available.

Our study has several strengths that contribute to the understanding of CP within the Chilean population. Firstly, the use of large, nationally representative samples from two distinct time points, along with a sampling method that ensures representativeness and generalizability to the Chilean population, supports the robustness and applicability of the findings. With 4,683 subjects in the 2009–2010 CNHS and 4,887 in the 2016–2017 CNHS, representing more than 12 million Chileans each, our sample sizes are significantly larger than those of previous studies (Bilbeny et al., 2018; Durán et al., 2023), allowing for more accurate and reliable estimates of CP prevalence. Secondly, the richness of the survey data allowed us to investigate a comprehensive set of associated factors, ranging from sociodemographic variables, nutrition status, and mental and cognitive health. Further, the addition of classic and advanced statistical techniques, such as machine learning algorithms like XGBoost, enhances the precision and predictive power of our findings.

This study has several limitations that should be acknowledged. First, its cross-sectional design precludes any inference of causal relationships between CP and the associated factors identified. Longitudinal studies will be necessary to establish temporal dynamics. Second, the potential underdiagnosis of cognitive impairment and dementia in the older adult population (Clark et al., 2005; Aranda et al., 2023) may have influenced the observed associations between cognitive functioning and CP. Diagnostic limitations in survey-based cognitive assessments and potential misclassification should be considered when interpreting these results. Third, the reliance on self-reported measures—including the presence of CP and comorbid conditions—and the lack of detailed information on medication use (e.g., analgesics or opioids) may introduce reporting and recall biases. Although the CNHS 2009–2010 and 2016–2017 cycles are the most recently available national datasets with relevant CP indicators, the current epidemiological landscape may have evolved, particularly in the wake of recent social, economic, and global events. Nevertheless, these datasets offer a valuable and representative baseline for understanding CP and its determinants in Chile. Future research should address these limitations through the use of longitudinal designs that allow for tracking changes in CP and its contributing factors over time. Improving the accuracy of cognitive assessments and expanding data collection to include additional contextual, behavioral, and clinical variables—such as healthcare access, pain attitudes, and treatment use—could further illuminate the complex nature of CP. Such evidence would be critical for developing tailored, equity-focused public health interventions to reduce the burden of CP, especially among socioeconomically vulnerable populations.

This study represents the first comprehensive characterization of CP prevalence and its associated factors using nationally representative data in Chile. Beyond identifying demographic, socioeconomic, and health-related correlates, our findings highlight the importance of incorporating the broader psychosocial and functional context in the assessment and management of CP. In clinical settings, this involves not only considering pain intensity or anatomical distribution, but also recognizing co-occurring conditions such as mobility limitations, mood disturbances, and cognitive changes that may shape the lived experience of pain. Similarly, in patients presenting with these comorbidities, early assessment of pain may contribute to a more complete clinical picture. These insights point to the need for future research that examines the dynamic interplay between CP and its associated factors over time and informs the development of care strategies that are responsive to the contextual and structural determinants shaping the burden of CP in aging populations.

## Data Availability

The datasets presented in this study can be found in online repositories. The names of the repository/repositories and accession number(s) can be found below: https://github.com/carolina-gonzalez-silva/Chronic-Pain-in-the-Chilean-population-prevalence-and-associated-factors.

## References

[B1] Adkins‐Jackson P. B. George K. M. Besser L. M. Hyun J. Lamar M. Hill‐Jarrett T. G. (2023). The structural and social determinants of Alzheimer’s disease related dementias. Alzheimer’s and Dementia 19 (7), 3171–3185. 10.1002/alz.13027 PMC10599200 37074203

[B2] Aguilar-Latorre A. Asensio-Martínez Á. Oliván-Blázquez B. Álvarez-Bueno C. Cavero-Redondo I. Lionis C. (2023). Association between sense of coherence and depression in patients with chronic pain: a systematic review and meta-analysis. PLos One 18 (1), e0279959. 10.1371/journal.pone.0279959 36630397 PMC9833581

[B3] Andrade C. (2015). Understanding relative risk, odds ratio, and related terms: as simple as it can get. J. Clin. Psychiatry 76 (07), e857–e861. 10.4088/JCP.15f10150 26231012

[B4] Aranda M. P. Marquez D. X. Gallagher-Thompson D. Pérez A. Rojas J. C. Hill C. V. (2023). A call to address structural barriers to Hispanic/Latino representation in clinical trials on Alzheimer’s disease and related dementias: a micro-meso-macro perspective. Alzheimer’s and Dementia Transl. Res. and Clin. Interventions 9 (2), e12389. 10.1002/trc2.12389 PMC10242183 37287471

[B5] Arango-Dávila C. A. Rincón-Hoyos H. G. (2018). Depressive disorder, anxiety disorder and chronic pain: multiple manifestations of a common clinical and pathophysiological core. Rev. Colomb. Psiquiatr. (Engl. Ed.) 47 (1), 46–55. 10.1016/j.rcp.2016.10.007 29428122

[B6] Asmundson G. J. Katz J. (2009). Understanding the co‐occurrence of anxiety disorders and chronic pain: state‐of‐the‐art. Depress. anxiety 26 (10), 888–901. 10.1002/da.20600 19691031

[B7] Baez S. Alladi S. Ibanez A. (2023). Global South research is critical for understanding brain health, ageing and dementia. Clin. and Transl. Med 13 (11), e1486. 10.1002/ctm2.1486 37987144 PMC10660824

[B8] Bartoskova Polcrova A. Dalecka A. Szabo D. Gonzalez Rivas J. P. Bobak M. Pikhart H. (2023). Social and environmental stressors and cardiometabolic risk. Eur. J. Public Health 33 (Suppl. ment_2), ckad160–1258. 10.1093/eurpub/ckad160.1258 PMC11187061 38898083

[B9] Befus D. R. Irby M. B. Coeytaux R. R. Penzien D. B. (2018). A critical exploration of migraine as a health disparity: the imperative of an equity-oriented, intersectional approach. Curr. pain headache Rep. 22, 79–8. 10.1007/s11916-018-0731-3 30291549

[B10] Bilbeny N. Miranda J. P. Eberhard M. E. Ahumada M. Méndez L. Orellana M. E. (2018). Survey of chronic pain in Chile – prevalence and treatment, impact on mood, daily activities and quality of life. Scand. J. Pain 18 (3), 449–456. 10.1515/sjpain-2018-0076 29886456

[B11] Bonathan C. Hearn L. Williams ACDC (2013). Socioeconomic status and the course and consequences of chronic pain. Pain Manage 3 (3), 159–162. 10.2217/pmt.13.18 24654756

[B12] Cao S. Fisher D. W. Yu T. Dong H. (2019). The link between chronic pain and Alzheimer’s disease. J. Neuroinflammation 16 (1), 204. 10.1186/s12974-019-1608-z 31694670 PMC6836339

[B13] Castañeda Morales V. M. Jiménez Garduño A. M. Escárcega M. V. Sánchez Velázquez L. D. Becerra Laparra I. (2015). Association between chronic pain and frailty in mexican elders. J. Frailty Aging, 1–3. 10.14283/jfa.2015.71 26980370

[B14] Chen J. Wang X. Xu Z. (2023). The relationship between chronic pain and cognitive impairment in the elderly: a review of current evidence. JPR 16, 2309–2319. 10.2147/JPR.S416253 PMC10335316 37441267

[B15] Chen T. Guestrin C. (2016). “XGBoost: a scalable tree boosting system,” in En: Proceedings of the 22nd ACM SIGKDD International Conference on Knowledge Discovery and Data Mining (California USA: ACM), 785–794. 10.1145/2939672.2939785

[B16] Clark C. M. DeCarli C. Mungas D. Chui H. I. Higdon R. Nuñez J. (2005). Earlier onset of alzheimer disease symptoms in latino individuals compared with anglo individuals. Archives Neurology 62 (5), 774–778. 10.1001/archneur.62.5.774 15883265

[B17] Cohen S. P. Vase L. Hooten W. M. (2021). Chronic pain: an update on burden, best practices, and new advances. Lancet 397 (10289), 2082–2097. 10.1016/S0140-6736(21)00393-7 34062143

[B18] Costigan M. Scholz J. Woolf C. J. (2009). Neuropathic pain: a maladaptive response of the nervous system to damage. Annu. Rev. Neurosci. 32 (1), 1–32. 10.1146/annurev.neuro.051508.135531 19400724 PMC2768555

[B19] Deckers K. Cadar D. van Boxtel M. P. Verhey F. R. Steptoe A. Köhler S. (2019). Modifiable risk factors explain socioeconomic inequalities in dementia risk: evidence from a population-based prospective cohort study. J. Alzheimer’s Dis. 71 (2), 549–557. 10.3233/JAD-190541 31424404 PMC6839472

[B20] Deguen S. Amuzu M. Simoncic V. Kihal-Talantikite W. (2022). Exposome and social vulnerability: an overview of the literature review. Int. J. Environ. Res. Public Health 19 (6), 3534. 10.3390/ijerph19063534 35329217 PMC8955941

[B21] Dellaroza M. S. G. Pimenta CADM Duarte Y. A. Lebrão M. L. (2013). Dor crônica em idosos residentes em São Paulo, Brasil: prevalência, características e associação com capacidade funcional e mobilidade (Estudo SABE). Cad. Saúde Pública 29 (2), 325–334. 10.1590/s0102-311x2013000200019 23459818

[B22] De Moraes Vieira É. B. Garcia J. B. S. Da Silva A. A. M. Mualem Araújo R. L. T. Jansen R. C. S. (2012). Prevalence, characteristics, and factors associated with chronic pain with and without neuropathic characteristics in São Luís, Brazil. J. Pain Symptom Manag. 44 (2), 239–251. 10.1016/j.jpainsymman.2011.08.014 22871508

[B23] Durán J. Tejos-Bravo M. Cid V. Ferreccio C. Calvo M. (2023). Chronic pain in Chile: first prevalence report of noncancer chronic pain, fibromyalgia, and neuropathic pain and its associated factors. Pain 164 (8), 1852–1859. 10.1097/j.pain.0000000000002886 36893316

[B24] Durán J. Zitko P. Barrios P. Margozzini P. (2021). Chronic musculoskeletal pain and chronic widespread pain in Chile: prevalence study performed as part of the national health survey. J. Clin. Rheumatol. 27 (6S), S294–S300. 10.1097/RHU.0000000000001642 33252393

[B25] Edwards R. R. Campbell C. Schreiber K. L. Meints S. Lazaridou A. Martel M. O. (2022). Multimodal prediction of pain and functional outcomes 6 months following total knee replacement: a prospective cohort study. BMC Musculoskelet. Disord. 23 (1), 302. 10.1186/s12891-022-05239-3 35351066 PMC8966339

[B26] Fittipaldi S. Migeot J. Ibanez A. (2024). Socioeconomic disparities harm social cognition. Trends Cognitive Sci. 28, 386–387. 10.1016/j.tics.2023.12.005 PMC11195298 38185605

[B27] Folstein M. F. Folstein S. E. McHugh P. R. (1975). “Mini-mental state”. A practical method for grading the cognitive state of patients for the clinician. J. Psychiatric Res. 12 (3), 189–198. 10.1016/0022-3956(75)90026-6 1202204

[B28] Gündoğdu S. (2023). Efficient prediction of early-stage diabetes using XGBoost classifier with random forest feature selection technique. Multimed. Tools Appl. 82 (22), 34163–34181. 10.1007/s11042-023-15165-8 PMC10043839 37362660

[B29] Ibanez A. Melloni L. Świeboda P. Hynes W. Ikiz B. Ayadi R. (2024). Neuroecological links of the exposome and one health. Neuron 112 (12), 1905–1910. 10.1016/j.neuron.2024.04.016 38723637 PMC11189719

[B30] Icaza M. G. Albala C. (1999). “Minimental State Examination: Análisis estadístico del estudio de demencia en Chile para validar una versión abreviada,” in Investigacones en Salud Pública: Documentos Técnicos, publicado por la Organización Panamericana de la Salud. Washington, DC.

[B31] Innes K. E. Sambamoorthi U. (2020). The potential contribution of chronic pain and common chronic pain conditions to subsequent cognitive decline, new onset cognitive impairment, and incident dementia: a systematic review and conceptual model for future research. JAD 78 (3), 1177–1195. 10.3233/JAD-200960 33252087 PMC7992129

[B32] Jennifer S. Brady B. R. Ibrahim M. M. Herder K. E. Wallace J. S. Padilla A. R. (2024). Co-occurrence of chronic pain and anxiety/depression symptoms in US adults: prevalence, functional impacts, and opportunities. Pain 165 (3), 666–673. 10.1097/j.pain.0000000000003056 37733475 PMC10859853

[B33] Kalaria R. Maestre G. Mahinrad S. Acosta D. M. Akinyemi R. O. Alladi S. (2024). The 2022 symposium on dementia and brain aging in low‐ and middle‐income countries: highlights on research, diagnosis, care, and impact. Alzheimer’s and Dementia, 13836. 10.1002/alz.13836 PMC11180946 38696263

[B34] Kawai K. Kawai A. T. Wollan P. Yawn B. P. (2017). Adverse impacts of chronic pain on health-related quality of life, work productivity, depression and anxiety in a community-based study. Fam. Pract. 34 (6), 656–661. 10.1093/fampra/cmx034 28444208 PMC6260800

[B35] Lasalvia P. Gil-Rojas Y. Rosselli D. (2022). Burden of disease of chronic pain in Colombia. Expert Rev. Pharmacoeconomics and Outcomes Res. 22 (8), 1261–1267. 10.1080/14737167.2022.2125872 36106600

[B36] Leão F. K. A. S. Bastos TRPD Andrade D. C. D. Silva A. M. Appolinario J. C. Teixeira M. J. (2016). Prevalence of chronic pain in a metropolitan area of a developing country: a population-based study. Arq. Neuro-Psiquiatr 74 (12), 990–998. 10.1590/0004-282x20160156 27991997

[B37] Leme M. de O. P. Yuan S. L. K. Magalhães M. O. de Meneses S. F. Marques A. P. (2019). Pain and quality of life in knee osteoarthritis, chronic low back pain and fibromyalgia: a comparative cross-sectional study. Reumatismo 71 (2), 68–74. 10.4081/reumatismo.2019.1104 31309776

[B38] Lerman S. F. Rudich Z. Brill S. Shalev H. Shahar G. (2015). Longitudinal associations between depression, anxiety, pain, and pain-related disability in chronic pain patients. Psychosom. Med. 77 (3), 333–341. 10.1097/PSY.0000000000000158 25849129

[B39] Leyva E. O. Bockos I. F. Vela Barba C. L. Aldazabal D. A. Vitorino C. E. García-Mostajo J. A. (2023). Pain prevalence and chronicity in a developing country in Latin America: a population-based survey in Lima, Peru. Pain Manag. 13 (1), 45–59. 10.2217/pmt-2022-0061 36264070

[B40] Maia Costa Cabral D. Sawaya Botelho Bracher E. Dylese Prescatan Depintor J. Eluf-Neto J. (2014). Chronic pain prevalence and associated factors in a segment of the population of São Paulo city. J. Pain 15 (11), 1081–1091. 10.1016/j.jpain.2014.07.001 25038400

[B41] Martineau D. B. Fornasini M. Suárez D. Paz M. Valarezo C. Loor E. (2023). Epidemiology of non-oncological high-impact chronic pain in Ecuadorian adults in 2022. Pain Manag. 13 (12), 689–699. 10.2217/pmt-2023-0055 38193278

[B42] Martínez N. T. Gómez-Restrepo C. Ramírez S. Rodríguez M. N. (2016). Prevalencia de trastornos del afecto y de ansiedad en personas con condiciones crónicas. Resultado de la Encuesta Nacional de Salud Mental Colombia 2015. Rev. Colomb. Psiquiatr. 45, 141–146. 10.1016/j.rcp.2016.06.001 27993249

[B43] Meghani S. H. Polomano R. C. Tait R. C. Vallerand A. H. Anderson K. O. Gallagher R. M. (2012). Advancing a national agenda to eliminate disparities in pain care: directions for health policy, education, practice, and research. Pain enero 13 (1), 5–28. 10.1111/j.1526-4637.2011.01289.x 22142450

[B44] Migeot J. Panesso C. Duran-Aniotz C. Ávila-Rincón C. Ochoa C. Huepe D. (2024). Allostasis, health, and development in Latin America. Neurosci. and Biobehav. Rev. 162, 105697. 10.1016/j.neubiorev.2024.105697 38710422 PMC11162912

[B45] Milani S. A. Bell T. R. Crowe M. Pope C. N. Downer B. (2023). Increasing pain interference is associated with cognitive decline over four years among older Puerto Rican adults. Journals Gerontology Ser. A 78 (6), 1005–1012. 10.1093/gerona/glac141 PMC10235200 35881065

[B46] Milani S. A. Sanchez C. Kuo Y. Downer B. Al Snih S. Markides K. S. (2024). Pain and incident cognitive impairment in very old Mexican American adults. J Am. Geriatrics Soc. 72 (1), 226–235. 10.1111/jgs.18618 PMC10842321 37794825

[B47] Ministry of Health (MINSAL) (2010). National health survey 2009–2010: first report of results. Available online at: https://epi.minsal.cl/wp-content/uploads/2016/05/presentacioÌ_nENS2010final-20-de-enero.pdf.

[B48] Ministry of Social Development and Family (2018). “Older adults results,” in CASEN survey 2017 (Chile: Government of Chile), 53. Available online at: https://observatorio.ministeriodesarrollosocial.gob.cl.

[B49] Ministry of Health (MINSAL) (2017). National health survey 2016–2017: main results. Available online at: https://redsalud.ssmso.cl/wp-content/uploads/2018/02/ENS-2016-17_PRIMEROS-RESULTADOS-ilovepdf-compressed.pdf.

[B50] Morete M. C. Solano J. Boff M. Jacob-Filho W. Ashmawi H. (2018). Resilience, depression, and quality of life in elderly individuals with chronic pain followed up in an outpatient clinic in the city of São Paulo, Brazil. JPR 11, 2561–2566. 10.2147/JPR.S166625 PMC6209073 30464576

[B51] Moriarty O. McGuire B. E. Finn D. P. (2011). The effect of pain on cognitive function: a review of clinical and preclinical research. Prog. Neurobiol. 93 (3), 385–404. 10.1016/j.pneurobio.2011.01.002 21216272

[B52] Müller A. C. Guido S. (2016). Introduction to machine learning with Python: a guide for data scientists. Sebastopol, CA: O’Reilly Media, Inc.

[B53] Nahin R. L. DeKosky S. T. (2020). Comorbid pain and cognitive impairment in a nationally representative adult population: prevalence and associations with health status, health care utilization, and satisfaction with care. Clin. J. Pain 36 (10), 725–739. 10.1097/AJP.0000000000000863 32740305 PMC9396625

[B54] Nuttall F. Q. (2015). Body Mass Index: obesity, BMI, and health A critical review. Nutr. Today 50 (3), 117–128. 10.1097/NT.0000000000000092 27340299 PMC4890841

[B55] Oliveira AMBD Teixeira DSDC Menezes F. D. S. Marques A. P. Duarte YADO Casarotto R. A. (2023). Socioeconomic and sex inequalities in chronic pain: a population-based cross-sectional study. PLoS ONE 18 (5), e0285975. 10.1371/journal.pone.0285975 37228121 PMC10212187

[B56] Paz M. G. D. Souza LAFD Tatagiba BDSF Serra J. R. D. Moura L. A. D. Barbosa M. A. (2021). Factors associated with quality of life of older adults with chronic pain. Rev. Bras. Enferm. 74 (Suppl. 2), e20200554. 10.1590/0034-7167-2020-0554 34037193

[B57] Pereira F. G. França M. H. Paiva MCAD Andrade L. H. Viana M. C. (2017). Prevalence and clinical profile of chronic pain and its association with mental disorders. Rev. saúde pública 51, 96. 10.11606/S1518-8787.2017051007025 29166447 PMC5676726

[B58] Pereira L. V. Vasconcelos P. P. D. Souza L. A. F. Pereira G. D. A. Nakatani A. Y. K. Bachion M. M. (2014). Prevalence and intensity of chronic pain and self-perceived health among elderly people: a population-based study. Rev. Latino-Am Enferm. 22 (4), 662–669. 10.1590/0104-1169.3591.2465 PMC4292652 25296151

[B59] Phelps C. E. Navratilova E. Porreca F. (2021). Cognition in the chronic pain experience: preclinical insights. Trends Cognitive Sci. 25 (5), 365–376. 10.1016/j.tics.2021.01.001 PMC8035230 33509733

[B60] Prado P. Medel V. Gonzalez-Gomez R. Sainz-Ballesteros A. Vidal V. Santamaría-García H. (2023). The BrainLat project, a multimodal neuroimaging dataset of neurodegeneration from underrepresented backgrounds. Sci. Data 10 (1), 889. 10.1038/s41597-023-02806-8 38071313 PMC10710425

[B61] Python Software Foundation (2024). Python programming language. Available online at: https://www.python.org/.

[B62] Raja S. N. Carr D. B. Cohen M. Finnerup N. B. Flor H. Gibson S. (2020). The revised International Association for the Study of Pain definition of pain: concepts, challenges, and compromises. Pain 161 (9), 1976–1982. 10.1097/j.pain.0000000000001939 32694387 PMC7680716

[B63] Sagi O. Rokach L. (2018). Ensemble learning: a survey. WIREs Data Min and Knowl 8 (4), e1249. 10.1002/widm.1249

[B64] Santamaria-Garcia H. Sainz-Ballesteros A. Hernandez H. Moguilner S. Maito M. Ochoa-Rosales C. (2023). Factors associated with healthy aging in Latin American populations. Nat septiembre 29 (9), 2248–2258. 10.1038/s41591-023-02495-1 PMC10504086 37563242

[B65] Schmiesing A. Liang Y. Turner B. J. (2022). Association of nonpharmacologic chronic pain management with function in a low‐income population: evidence from a survey of a sample of Latinos from five states. PM&R 14 (11), 1343–1350. 10.1002/pmrj.12701 34464031

[B66] Serra J. R. D. Souza L. A. F. Paz M. G. D. Tatagiba BDSF Pereira L. V. (2021). Sex differences in coping strategies based on chronic pain intensity among older adults. J. Gerontol. Nurs. 47 (10), 30–36. 10.3928/00989134-20210908-05 34590979

[B67] Shahriari B. Swersky K. Wang Z. Adams R. P. De Freitas N. (2016). Taking the human out of the loop: a review of bayesian optimization. Proc. IEEE 104 (1), 148–175. 10.1109/jproc.2015.2494218

[B68] Silvestre B. A. Miotto L. P. Gramani-Say K. Barbosa M. H. Hortense P. (2023). Chronic pain and associated factors in remote work during the COVID-19 pandemic in Brazil. Rev. Bras. Enferm. 76 (Suppl. 1), e20230012. 10.1590/0034-7167-2023-0012 PMC10695055 38055431

[B69] Souza J. B. D. Grossmann E. Perissinotti D. M. N. Oliveira Junior J. O. D. Fonseca PRBD Posso I. D. P. (2017). Prevalence of chronic pain, treatments, perception, and interference on life activities: Brazilian population-based survey. Pain Res. Manag. 2017, 1–9. 10.1155/2017/4643830 PMC5634600 29081680

[B70] Superintendencia de Salud (2025). Explicit health Guarantees (GES). Chile: Government of Chile. Available online at: https://www.superdesalud.gob.cl (Accessed June 4, 2025).

[B71] Szumilas M. (2010). Explaining odds ratios. J. Can. Acad. Child. Adolesc. Psychiatry 19 (3), 227–229. 20842279 PMC2938757

[B72] Takahashi A. Kitamura K. Watanabe Y. Kobayashi R. Saito T. Takachi R. (2018). Epidemiological profiles of chronic low back and knee pain in middle-aged and elderly Japanese from the Murakami cohort. J. pain Res. 11, 3161–3169. 10.2147/JPR.S184746 30588068 PMC6296201

[B73] Treede R. D. Rief W. Barke A. Aziz Q. Bennett M. I. Benoliel R. (2019). Chronic pain as a symptom or a disease: the IASP classification of chronic pain for the international classification of diseases (ICD-11). Pain 160 (1), 19–27. 10.1097/j.pain.0000000000001384 30586067

[B74] Vélez J. C. Kovasala M. Collado M. D. Friedman L. E. Juvinao-Quintero D. L. Araya L. (2022). Pain, mood, and suicidal behavior among injured working adults in Chile. BMC Psychiatry 22 (1), 766. 10.1186/s12888-022-04391-3 36471330 PMC9724445

[B75] Wallace B. Varcoe C. Holmes C. Moosa-Mitha M. Moor G. Hudspith M. (2021). Towards health equity for people experiencing chronic pain and social marginalization. Int. J. Equity Health 20 (1), 53. 10.1186/s12939-021-01394-6 33531018 PMC7852178

[B76] Whitlock E. L. Diaz-Ramirez L. G. Glymour M. M. Boscardin W. J. Covinsky K. E. Smith A. K. (2017). Association between persistent pain and memory decline and dementia in a longitudinal cohort of elders. JAMA Intern Med. 177 (8), 1146–1153. 10.1001/jamainternmed.2017.1622 28586818 PMC5588896

[B77] Williams D. R. Mohammed S. A. (2009). Discrimination and racial disparities in health: evidence and needed research. J. Behav. Med. 32 (1), 20–47. 10.1007/s10865-008-9185-0 19030981 PMC2821669

[B78] Yong R. J. Mullins P. M. Bhattacharyya N. (2022). Prevalence of chronic pain among adults in the United States. Pain 163 (2), e328–e332. 10.1097/j.pain.0000000000002291 33990113

[B79] Zimmer Z. Fraser K. Grol-Prokopczyk H. Zajacova A. (2022). A global study of pain prevalence across 52 countries: examining the role of country-level contextual factors. Pain 163 (9), 1740–1750. 10.1097/j.pain.0000000000002557 35027516 PMC9198107

[B80] Zitko P. Bilbeny N. Balmaceda C. Abbott T. Carcamo C. Espinoza M. (2021). Prevalence, burden of disease, and lost in health state utilities attributable to chronic musculoskeletal disorders and pain in Chile. BMC Public Health 21 (1), 937. 10.1186/s12889-021-10953-z 34001042 PMC8130395

