# Supplementary material for: Chronic pain in the Chilean population: risk factors prevalence and cognitive associations
Source: Front Aging. 2025 Jul 22;6:1548667. doi: 10.3389/fragi.2025.1548667 (PMC12321896; doi:10.3389/fragi.2025.1548667)
Supplement: Supplementary file 1 [file DataSheet2.pdf]

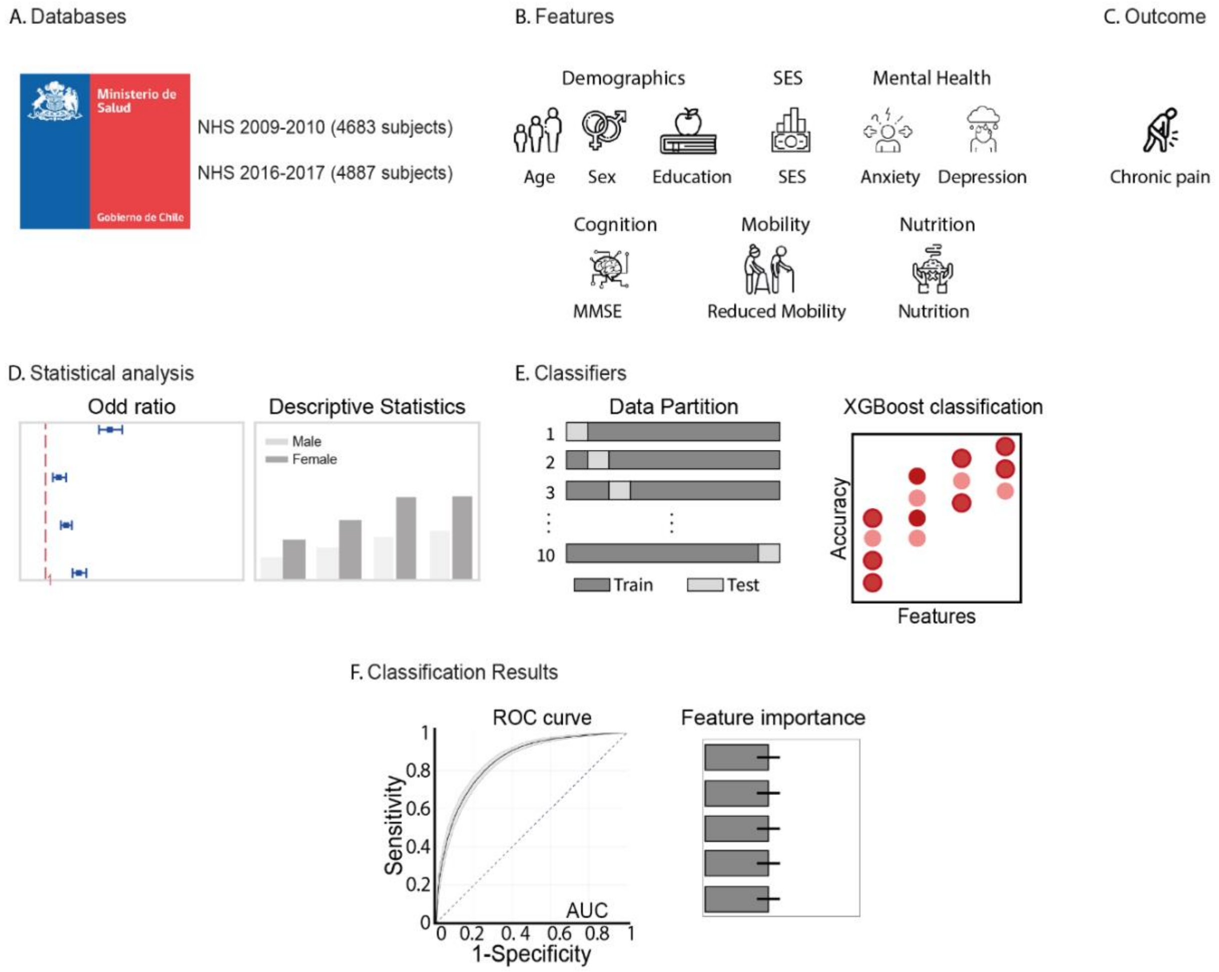

A. Databases
Ministerio de Salud
Gobierno de Chile
NHS 2009-2010 (4683 subjects)
NHS 2016-2017 (4887 subjects)
B. Features
Demographics
Age
Sex
Education
SES
SES
Mental Health
Anxiety
Depression
Cognition
MMSE
Mobility
Reduced Mobility
Nutrition
Nutrition
C. Outcome
Chronic pain
D. Statistical analysis
Odd ratio
Descriptive Statistics
Male
Female
E. Classifiers
Data Partition
1
2
3
10
Train
Test
XGBoost classification
Accuracy
Features
F. Classification Results
ROC curve
Sensitivity
1-Specificity
AUC
0 0.2 0.4 0.6 0.8 1
Feature importance
