# Supplementary material for: Chronic pain in the Chilean population: risk factors prevalence and cognitive associations
Source: Front Aging. 2025 Jul 22;6:1548667. doi: 10.3389/fragi.2025.1548667 (PMC12321896; doi:10.3389/fragi.2025.1548667)
Supplement: Supplementary file 2 [file DataSheet1.pdf]

Supplementary Figure 1. (A) Databases: We utilized the Chilean Health Surveys from 2009-2010 and 2016-2017, comprising 4683 and 4887 subjects, respectively. These subjects are under 80 years old and reported no history of cancer. **(B)** Features: Demographic variables (age, sex, and years of education), socioeconomic status (SES), mood disorders (anxiety and depression), cognition, mobility reduction, and nutrition were extracted as features. **(C)** Outcome: The target variable in our study was CP, defined for participants who reported pain for more than 90 days. **(D)** Statistical Analysis: Various statistical analyses were performed, including odds ratio calculation, and descriptive statistics to explore the association between features and the outcome. **(E)** Classifier: XGBoost was applied for predicting CP, employing data partitioning with an 80% training sample and a 20% testing set, repeated 10 times (k = 10 repetitions). **(F)** Classification Results: We reported the ROC curve of the classification, along with feature importance. Analyses were conducted separately for the 2009-2010 and 2016-2017 CNHS.

Supplementary Table 1. CP proportions across age ranges for the CNHS surveys conducted in 2009-2010 and 2016-2017.

	**CNHS 2009-2010**		**CNHS 2016-2017**	
**Age range (years)**	**Proportion**	**95% conf. interval**	**Proportion**	**95% conf. interval**
15-24	0.25	0.21-0.30	0.13	0.08-0.21
25-44	0.42	0.38-0.46	0.25	0.18-0.33
45-64	0.54	0.50-0.58	0.38	0.31-0.46
65-80	0.59	0.52-0.66	0.42	0.31-0.53

Supplementary Table 2. Prevalence of CP within age ranges, divided by single or multi-pain, for the CNHS surveys of 2009-2010 and 2016-2017.

		**CNHS 2009-2010**		**CNHS 2016-2017**	
**Age range (years)**	**Pain**	**Proportion**	**95% conf. interval**	**Proportion**	**95% conf. interval**
15-24	Single	0.58	0.45-0.70	1	0
	Multi	0.74	0.62-0.83	0.69	0.37-0.89
25-44	Single	0.73	0.63-0.82	0.30	0.10-0.62
	Multi	0.76	0.68-0.83	0.89	0.78-0.95
45-64	Single	0.76	0.67-0.83	0.79	0.53-0.92
	Multi	0.92	0.87-0.95	0.82	0.72-0.90
65-80	Single	0.82	0.63-0.92	0.70	0.28-0.94
	Multi	0.97	0.94-0.99	0.90	0.79-0.95

Supplementary Table 3. Percentage breakdown of pain by body region for the 2009-2010 and 2016-2017 CNHS surveys.

Body zone	CNHS 2009-2010	CNHS 2016-2017
Knee	51.17	58.88
Back down	31.39	45.96
Shoulder	27.95	40.98
Wrist	25.15	35.66
Fingers	24.69	41.60
Ankle	23.77	33.24
Hips	22.86	34.76
Back up	20.15	36.14
Elbow	16.48	22.94
Neck	13.81	24.95
Toes	11.89	19.14
Others	12.02	6.70

Supplementary Table 4. Proportions of CP among individuals aged 60 and older, segmented by pain type, for the CNHS surveys conducted in 2009-2010 and 2016-2017.

			CNHS 2009-2010		CNHS 2016-2017	
**Cognition**	**Sex**	**Pain**	**Proportion**	**95% conf. interval**	**Proportion**	**95% conf. interval**
No-cognitive impairment	Total	Single	0.80	0.64-0.90	0.79	0.53-0.92
		Multi	0.95	0.89-0.98	0.89	0.77-0.95
	Male	Single	0.78	0.54-0.92	0	-
		Multi	0.90	0.71-0.97	0.90	0.58-0.98
	Female	Single	0.82	0.62-0.93	0.82	0.49-0.96
		Multi	0.98	0.95-0.99	0.88	0.73-0.95
Cognitive impairment	Total	Single	0.79	0.60-0.91	0.20	0.01-0.83
		Multi	0.96	0.82-0.99	0.84	0.50-0.97
	Male	Single	0.80	0.43-0.96	0	-
		Multi	0.90	0.63-0.98	0.71	0.19-0.96
	Female	Single	0.79	0.53-0.93	0.20	0.73-0.95
		Multi	1	-	0.88	0.43-0.99
